# Medical Items

**Published:** 1913-06

**Authors:** 


### MEDICAL ITEMS

### CHRONIC CATARRHAL DISEASES.

Chronic catarrh never fails to indicate general constitutional debility. Local treatment is always desirable but for permanent results efforts must be directed toward promoting general functional activity throughout the body, and a general increase of systemic vitality. The notable capacity of Gray's Glycerine Tonic Comp. in this direction readily accounts for the gratifying results that can be accomplished through its use in the treatment of all chronic catarrhal affections, but especially those of the gastro-intestinal and respiratory tract. The particularly gratifying features in the results accomplished by Gray's Glycerine Tonic Comp. are their substantial and permanent character. This is naturally to be expected since they are brought about through restoring the physiologic balance of the whole organism.

